# Supplementary material for: IGF-1 and IGF-2 as Molecules Linked to Causes and Consequences of Obesity from Fetal Life to Adulthood: A Systematic Review
Source: Int J Mol Sci. 2024 Apr 2;25(7):3966. doi: 10.3390/ijms25073966 (PMC11012406; doi:10.3390/ijms25073966)
Supplement: Supplementary file 1 [file ijms-25-03966-s001.zip › ijms-2903030-supplementary.pdf]

Table S1. IGF-1 and IGF-2 values (ng/mL) during child development.

Reference	Study group	Country	Age (mean; years, unless marked differently)	Age (range; years, unless marked differently)	Pubertal status	IGF-1 (mean; ng/mL)	IGF-1 (range; ng/mL)	IGF-2 (ng/mL)
4	574 obese children	Italy	11.34			353.5 - 363.9		656.6- 704.7
19	269 infants born to non-obese mothers (1), 130 infants born to obese mothers (2), heavier than (1) (p=0,020)	Denmark	9-months	9-months		48.1 (1), 34.7 (2)	35.4-60.2 (1), 26.0- 46.4 (2)	
22	577 infants born to obese mothers	UK	cord blood			45.0		496.0
2	28 obese children (1), 34 healthy (2) children	Poland	13.7 (1), 11.8 (2)	4–17.8 (1), 4.3–16.9 (2)		299 (1), 231 (2)		51.95 (1), 107 (2)
24	611 normal weight (1), 50 obese (2) girls	India	11.7	5-18		368.9 (1), 332.2(2)	335.9–371.5 (1), 286.6–377.8 (2)	
26	84 obese (1), 43 non-obese (2) children	China	4-18			180.5 (1), 315 (2)	128.75–268.5 (1), 185–460 (2)	
27	78 obese (1), 30 non-obese (2) children	China	12.24 (1), 11.7 (2)			268.36 (1), 390.25 (2)		
28	53 normal weight (1), 48 overweight or obese (2) children	Puerto Rico, USA	15.3 (1), 14.9 (2).	12-18		272.1 (1), 216.3 (2)	186.6-384.0 (1), 166.5-263.1 (2)	
30	2059 healthy children	Germany		0.14-18.89		peak in Tanner scale (TS) 4: boys: 387.4, girls: 336.3		
3	107 normal-weight (1), 81 overweight/obese (2) children	Germany	11.05 (1) 12.89 (2)			198.87 (1), 284.19 (2)	32.3-484.6 (1), 38.5-549.4 (2)	
31	33 normal-weight (1), 28 obese (2) children	Montenegro		9-12		177.4 (1), 227.45 (2)	121.9-239.2 (1), 160.1-426.3 (2)	
32	23 obese and overweight (1), 11 normal-weight (2) children	Italy		3-10	prepubertal	205.27 (1), 155.67 (2)		

34	24 non-obese (1), 43 obese (2) children	Spain		3-14		86.7 (1),<br>165 (2)	58.7-154 (1),<br>129.2-272.7 (2)
35	2341 normal weight (1),<br>278 obese (2) children	China	8.6 (1),<br>9.4 (2)	2.0-17.6 (1),<br>2.4-15.6 (2)		207.2 (1),<br>281.1 (2)	25.0-405.0 (1),<br>31.9-796.0 (2)
36	45 obese (1),<br>20 normal-weight (2) children	Poland	7.8 (1),<br>7.7 (2)	4-10	prepubertal	147.0 (1),<br>132.3 (2)	120.8–179.6 (1),<br>100.0–151.2 (2)
37	61 normal-weight (1),<br>28 obese (2) children with idiopathic short stature	Poland	10.99			194.37 (1),<br>252.02 (2)	
38	231 non-obese (1),<br>229 non-obese (2) children	Denmark	9 months (1),<br>36 months (2)	9 months (1),<br>36 months (2)		47.8 (1),<br>72.6 (2)	33.8-63.7 (1),<br>44.2-108.0 (2) *
39	115 healthy children	Denmark	9 months (1),<br>18 months (2)	9 months (1),<br>18 months (2)		49.3 (1),<br>62.9 (2) *	
40	215 overweight, 661 obese children	Italy	10.6		prepubertal and pubertal	268.5	
42	20 obese (1),<br>20 non-obese (2) children	Italy	11.2 (1),<br>10.3 (2)			315.4 (1),<br>195.9 (2)	
43	61 obese children	USA	14.4		most in late puberty TS 4-5	321	
44	972 obese and non-obese children	Chile	7	7	prepubertal	182.25	
46	56 normal weight,<br>91 obese children	USA		3-18.4	prepubertal, in-puberty, postpubertal	normal weight children [TS; girls, boys]:<br>TS1; 177.0, 144.7<br>TS2; 242.5, 196.0<br>TS3; 295.5, 297.7<br>TS4; 429.0, 320.5<br>TS5; 415.0, 320.5<br>Obese children [TS; girls, boys]:<br>TS1; 187.5, 148.2<br>TS2; 191.3, 211.0<br>TS3; 299.5, 241.1<br>TS4; 430.0, 404.0<br>TS5; 339.0, 310.3	

47	633 under-, normal- and over-weight (including obese) children	Denmark		8-11 years	prepubertal and pubertal	girls: 211, boys: 180	
49	60 obese children	Germany	12.0		prepubertal and early pubertal T2-3	291	
50	943 children	Chile	6.7		prepubertal T1	184.5 (girls: 176.7, boys: 189.3) *	
51	2695 normal weight (1), 392 obese children (2)	Germany		0-20.3		195.6 (1), 236.1 (2)	20-908 (1), 38.5-624 (2)
55	105 overweight children	Germany	Girls: 10.3 and 11.3 (while entering puberty, one year later)<br>Boys: 11.9 and 12.9 (while entering puberty, one year later)		prepubertal and in entry to puberty	Girls: baseline 215, entry into puberty 377<br>Boys: baseline 218, entry into puberty 337	

* Own calculations based on given data.
